# Uncertainty in Ecohydrological Modeling in an Arid Region Determined with Bayesian Methods

**DOI:** 10.1371/journal.pone.0151283

**Published:** 2016-03-10

**Authors:** Junjun Yang, Zhibin He, Jun Du, Longfei Chen, Xi Zhu

**Affiliations:** Linze Inland River Basin Research Station, Chinese Ecosystem Research Network, Key Laboratory of Eco-hydrology of Inland River Basin, Cold and Arid Regions Environmental and Engineering Research Institute, Chinese Academy of Sciences, Lanzhou, China; Southwest University, CHINA

## Abstract

In arid regions, water resources are a key forcing factor in ecosystem circulation, and soil moisture is the critical link that constrains plant and animal life on the soil surface and underground. Simulation of soil moisture in arid ecosystems is inherently difficult due to high variability. We assessed the applicability of the process-oriented CoupModel for forecasting of soil water relations in arid regions. We used vertical soil moisture profiling for model calibration. We determined that model-structural uncertainty constituted the largest error; the model did not capture the extremes of low soil moisture in the desert-oasis ecotone (DOE), particularly below 40 cm soil depth. Our results showed that total uncertainty in soil moisture prediction was improved when input and output data, parameter value array, and structure errors were characterized explicitly. Bayesian analysis was applied with prior information to reduce uncertainty. The need to provide independent descriptions of uncertainty analysis (UA) in the input and output data was demonstrated. Application of soil moisture simulation in arid regions will be useful for dune-stabilization and revegetation efforts in the DOE.

## Introduction

Soil moisture is the single most important variable in studies of hydrology, ecology, and climate change [1] because it forms intermediate links between runoff and groundwater, and the atmosphere and groundwater. It is critical for the survival of the vegetation, and it controls the distribution of soil heat fluxes [2]. Soil moisture is a function of precipitation, interception, evapotranspiration, and runoff [3, 4], and has been a subject of extensive studies in mesic environments.

Especially at the desert-oasis ecotone (DOE), soil water provides plants with available, transpirable pool of water critical for the survival of the vegetation. Soil moisture is highly variable in semiarid and arid regions. Its estimation is imperative for hydrometeorological studies and water resources management [5, 6]. Soil moisture in these systems is determined by the interplay of surface and near-surface processes that are dependent on abiotic and biotic characteristics of the ecosystem. Therefore, a greater understanding of soil moisture can help improve the management of scarce water resources in arid systems, and the modeling of the hydrologic cycle, extreme precipitation events, and vegetation growth. However, simulation and prediction of soil moisture in arid and semiarid regions has received far less attention than that in mesic systems.

The advantage of modeling soil moisture is an improved ability to quantify and optimize this resource in semiarid and arid regions where it is vastly limiting. In addition, accurate modeling of temporal and spatial variation in soil moisture may be useful in improving the prediction capability of runoff models, and by-passing the need to conduct time-consuming measurements of soil moisture time series [7]. Lastly, it can offer insights into watershed function, and project future watershed response to management, changing climatic conditions and land use.

In modeling, uncertainty is an inherent component of the model, and uncertainty analysis (UA) is a necessary step in model application [8]. Uncertainty and global sensitivity analysis (GSA) are tools that can be used to evaluate model fitness and quantify uncertainties of input and output data, and parameter and model structure [9].

Excluding conventional uncertainties, several recent studies explored novel sources and aspects of uncertainties, such as those related to the various climate scenarios [10], calibration periods [11], model components containing general circulation models, model structures, downscaling techniques, and model parameters of climate change [12]. The treatment of uncertainty in hydrology has also progressed in the last few decades, especially around the parameter uncertainty [13]. Failure to understand and account for these uncertainties in hydrological modeling can have serious implications for water resources management performance [14, 15].

Aimed at addressing such uncertainties, there has been a growing interest in the development of methods for stochastic decision processes that explicitly confirm the uncertainty in a response of ecosystems [16]. The potential applicability of Bayesian methods in complex model optimization had been advanced by coupling with the new statistical theory (such as the Markov chain Monte Carlo algorithms) only a decade ago [17, 18]. In that, particular attention has been placed on the implementation of Bayesian methods that enable the consideration of model uncertainty, and the ability to improve or update model predictions [19]. For example, characterization of uncertainty in the distribution of parameters was based historically on literature, field observation, and expert judgment [20]. Advancement of Bayesian methods allows new insights into the covariance structure among parameters, and credible intervals for model outputs.

The issues of uncertainties in modeling are compounded by high spatial and temporal variability of soil moisture in arid and semiarid regions. Thus, several challenges are specific to efforts at simulating ecohydrological processes in these systems. First, ephemeral vegetation redistributes the water balance after extreme rainfall events. Second, drifting sand can easily change the texture of the surface sand. In addition, fast changes of soil moisture at the surface (0–10 cm soil depth), and minimal (about 2–3% cm^3^cm^-3^) soil moisture contents in deeper soil layers defy most of the hydrological process models. In short, in arid and semi-arid systems, characteristics of the environment itself constitute new sources of uncertainties in the modeling of soil water.

The objectives of this study were to investigate sources of uncertainty, and to assess feasibility of model calibration for an arid region. Specifically, (1) we asked if the current model can simulate extremely low soil moistures (2–3% cm^3^cm^-3^), (2) we analyzed which portion of uncertainty (input data, output data, parameter, or model structure) were the main factors in the soil moisture simulation for arid regions.

## Materials and Methods

### Study site

**Fig 1:**
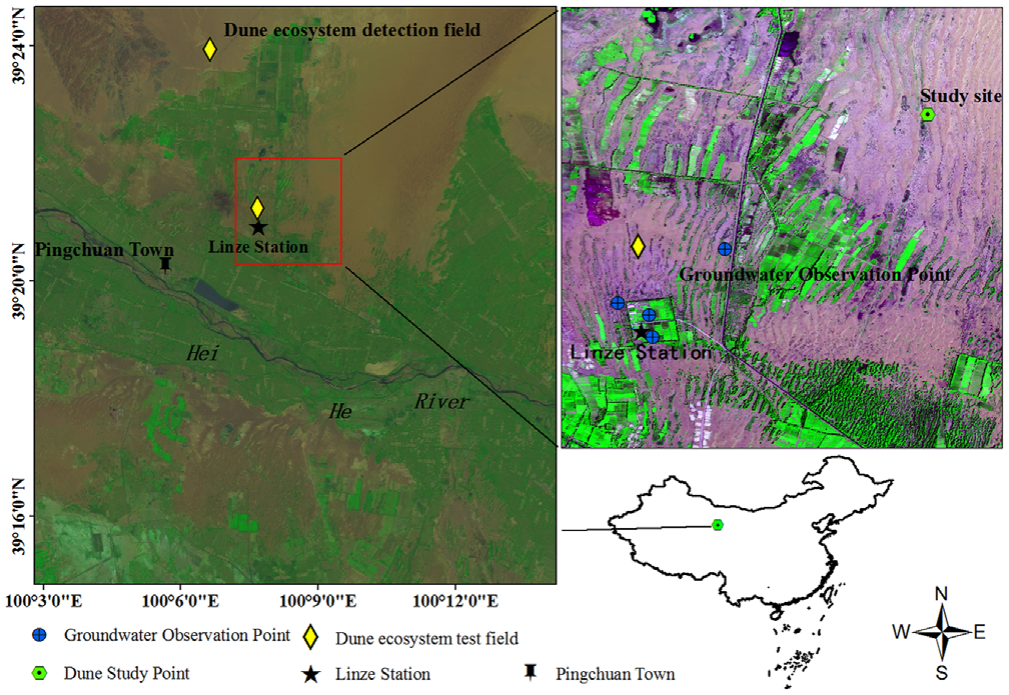
Locations of the dune study site, Heihe River, and the Linze National Station.

The study area is a DOE in the middle of the Heihe River basin, which is located in the northwestern part of China. The area is adjacent to residual dunes as well as the Gobi Desert [21], and constitutes a physical transition zone between irrigated farmland and the natural desert ecosystem, adding to its ecological fragility and climatic sensitivity [22]. According to climatic statistics from the Linze In-land River integrated station (39°21′N, 100°07′E), climate in the region is temperate arid with cold and dry winters and warm summers. The altitude is about 1367 m above sea level, and the mean annual precipitation is 117 mm, with a mean potential evaporation rate at about 20 times more than the annual precipitation. The mean annual air temperature is about 7.6°C with a recorded maximum of 39.1°C and a minimum of -27°C. The frost-free season lasts on average 105 days. The mean annual wind velocity is 3.2 m∙s^-1^ [21]. The study area and the weather station are 2.85 km apart and there is no difference in elevation (Fig 1).

### Field measurements and data preprocessing

#### Meteorological data

Meteorological measurements were obtained from the Linze station and included precipitation (mm/day), air temperature (°C), vapor pressure (Pa), global radiation (J/m^2^∙day), surface temperature (°C), and wind speed (m/s); all data used in this study were converted to daily values. In order to ensure the continuity of data used in the model calibration and validation, two years of observation data were chosen for this study. Thus, we had 397 days in total; the records were from May 17 to October 15, 2009, and from February 1 to September 30, 2013.

#### Soil moisture and temperature data

Verification data for the model contained soil moisture and soil temperature. First, the soil profile was measured in 8 layers within 100 cm depth, with a thickness of 10 cm for the first six, and 20 cm of the other two layers. Soil moisture probes were inserted at: 10, 20, 30, 40, 50, 60, 80 and 100 cm. Probes were inserted in a straight line vertically to try to imitate the transfer route of soil moisture. Soil temperature probes were located 5 cm away from the moisture probes at the same soil depths. Soil moisture probes used Time-Domain Reflectometer (Abbr.: TDR, CS645, manufactured by Campbell Scientific, rod length of 75 mm). Soil temperature data were acquired with a 109ss temperature sensor probe which was an ancillary equipment of the meteorological station, operating at half-hour frequencies. This probe had higher precision than capacitance sensors [23], and that was more suitable for our low soil moisture environment. Data were preprocessed to daily values (mean of the daily records) to match the meteorological data.

#### Forest inventory data

Natural vegetation at the site was composed of *Haloxylon ammodendron*, growing on top of the dunes, and *Nitraria sphaerocarpa*, found in the inter-dune lowland. The integrated meteorological station and TDR were located between the dunes. Therefore, in this study, we focused on *Nitraria sphaerocarpa*. The mean age of *Nitraria sphaerocarpa* was about 8 years, plant height 0.34 m, and the average root depth 2 times that of height. The growing season length averaged about 200 days, the distribution was very sparse random, and the mean cover was about 37% (Table 1).

**Table 1:** Characteristics of *Nitraria sphaerocarpa* investigated between 2006 and 2010.

Variable	Value	Variable	Value	Variable	Value
Age	8±4	Depth of groundwater (m)	3.0±0.5	Start day	100±7
Height (m)	0.34±0.15	Mean annual increment (cm)	5.0±2.3	Optimum day	269±4
Depth of root (m)	0.82±0.21	Mean cover (%)	37.0±12	End day	304±9

Values given are: mean ± std. deviation. ‘Start day’ is the day plant growth initiated since start of year; ‘Optimum day’ is the day of plant optimum growth and ‘End day’ is the day of plant growth cessation.

#### Soil texture and groundwater

The size (diameter) of soil particles was determined with Laser Particle Size Analyzer (LDSA) Mastersizer 2000 at the Institute of Soil and Water Conservation, CAS & MWR. Particles with a diameter above 2 mm were negligible and therefore ignored in the study. Sand occupied 85% of the soil components within the 100 cm depth, and somewhat less in the surface layer alone (about 83.8%); soil texture was disadvantageous to water storage (Table 2).

**Table 2:** Particle-size distribution of soil layers, Classification standard from the U. K.–ADAS, and ‘-’ means no record.

Layer (cm)	Clay (%)	Silt (%)	Sand (%)	Organic (%)
0–10	0.73±0.05	15.48±1.90	83.79±1.95	0.90±0.04
10–20	0.68±0.00	12.74±0.48	86.57±0.48	0.89±0.13
20–30	0.73±0.06	16.73±0.23	82.54±0.29	-
30–40	0.88±0.01	12.18±0.37	86.95±0.36	-
40–50	0.69±0.03	12.18±1.12	87.13±1.16	-
50–60	0.72±0.03	14.65±1.11	84.63±1.08	-
60–80	0.73±0.03	12.20±1.50	87.07±1.47	-
80–100	0.78±0.06	14.93±1.10	84.29±1.10	-

Depth of groundwater was derived from a groundwater observation well, which is 2 km away from the study site at a location with the same landscape pattern. The mean annual depth of groundwater from 2004 to 2010 was 2.87±0.15 m.

### Preprocessing of soil moisture data

**Fig 2:**
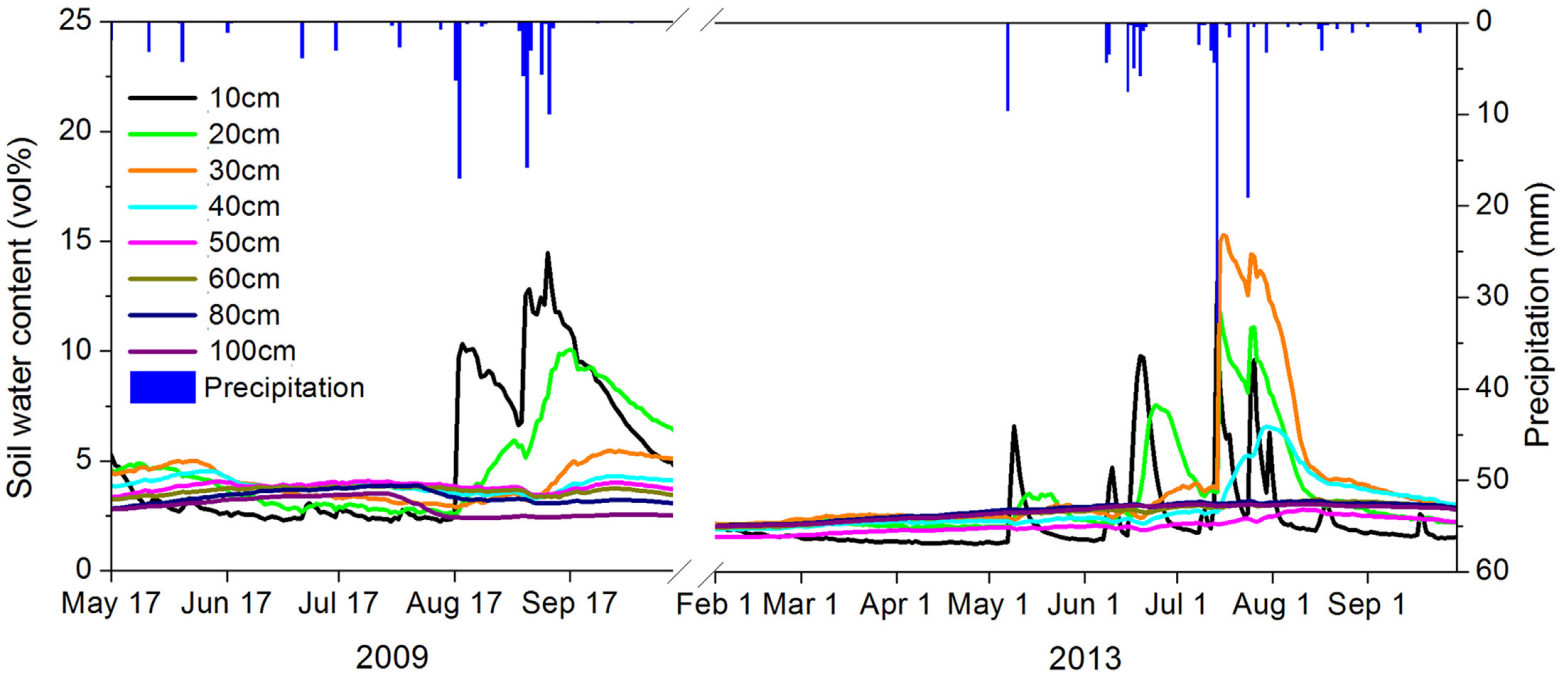
Soil water content dynamics and the corresponding precipitation processes between 2009 and 2013. Details and data used are described in the S1 File.

The observation period in this study spanned the vegetation growing season from the beginning of April to the end of October. Due to equipment malfunction in 2013, soil moisture data were not collected for 5 days, and soil temperature at 40 cm depth at all. Missing data were interpolated using the Cubic spline function in Matlab; thus, missing soil moisture was simulated from records obtained few days before and after, and soil temperature at 40 cm was simulated from the sequence at 30 and 50 cm depth records (Fig 2).

Parameters, which simulated the real situation in the calibration period, were used in the validation period. Our thought was that similar climate under the same parameters will generate better results. Second, a long calibration period is deemed the most robust approach for model calibration. For our data, we set 2013 as the calibration year, and 2009 as the validation year for the model.

### The CoupModel

We used the CoupModel which describes hydrological processes in the boreal region during the course of a year [8]. The model is strongly rooted in soil physics, and represents a coupling between the Richards Eq (1) for water flow and the Fourier Eq (2) for heat flow with an explicit numerical method in a one-dimensional domain.

Water flow in soil was assumed to be laminar and obey Darcy’s Law as generalised for unsaturated flow by Richards [24]:

$$q_{w}=-k_{w}\left(\frac{\partial \psi }{\partial z}-1\right)-D_{v}\frac{\partial c_{v}}{\partial z}+q_{bp}$$ (1)

Where *q*_*w*_ is the total water flow, *k*_*w*_ is unsaturated hydraulic conductivity, *D*_*v*_ is the diffusion coefficient in soil, *q*_*bp*_ is bypass flow in the macro-pores, *Ψ* is water tension, *z* is depth of soil, and *c*_*v*_ is the concentration of vapor in soil air.

Heat flow in the soil was represented using:

$$q_{h}=-k_{h}\frac{\partial T}{\partial z}+C_{w}Tq_{w}+L_{v}q_{v}$$ (2)

Where *h*, *v*, *w* are heat, vapor and liquid water, respectively; *q* is flux, *k* is conductivity, *T* is soil temperature, *C* is heat capacity, *L* is latent heat, and *z* is soil depth.

The empirical thermal conductivity function for non-frozen mineral soil was adapted from Kersten [25]:

$$k=0.143\left(a_{1}\text{log}\left(\frac{\theta }{\rho_{S}}\right)+a_{2}\right)10^{a_{3}\rho_{s}}$$ (3)

Where *a*_1_, *a*_2_, *a*_3_ are parameters and *ρ*_*s*_ is dry bulk soil density, and *θ* / *ρ*_*s*_ is equivalent to soil moisture by weight.

Vegetation was described in three ways. First, vegetation was regarded as an explicit big-leaf model, in which transpiration and soil evaporation were treated as common flow (soil evaporation is not calculated). Second, vegetation was one large leaf, and transpiration and soil evaporation were treated as separate flows; here, potential transpiration and transpiration were calculated with the Penman-Monteith Eq (4). Third, vegetation was represented as an array of plants, multiple canopies, and root systems. This was different from the explicit big-leaf model in that the presence of multiple plants made it possible to incorporate different stand characteristics in an area.

$$ET=\frac{\Delta (R_{n}-G)+\rho_{a}c_{p}\frac{e_{s}-e_{a}}{r_{a}}}{\Delta +\gamma \left(1+\frac{r_{s}}{r_{a}}\right)}$$ (4)

Where *R*_*n*_ is net radiation, *G* is the soil heat flux, *ρ*_*a*_ is mean air density at constant pressure, *c*_*p*_ is the specific heat of air, *e*_*s*_−*e*_*a*_ represents vapour pressure deficit of air, Δ represents the slope of the saturation vapour pressure temperature relationship, *γ* is the psychrometric constant, and *r*_*a*_, *r*_*s*_ are the aerodynamic resistances and surface.

### Soil water balance model for water fluxes

The water balance in the model was expressed by Eq (5).

$$S_{td}=I_{a}+P+P_{ad}-E_{ag}-T_{a}-E_{ai}-R_{as}-D_{a}$$ (5)

The values for irrigation (*I*_*a*_), deep percolation (*P*_*ad*_), surface runoff (*R*_*as*_), drainage (*D*_*a*_), ground evaporation (*E*_*ag*_), transpiration (*T*_*a*_), and interception evaporation (*E*_*ai*_), all accumulated over the study period, were set to 0, as appropriate for arid regions, and the equation was simplified to:

$$S_{td}=P-E_{st}-E_{ai}$$ (6)

Where: *S*_*td*_ was total difference storage, *P* precipitation, and *E*_*st*_ soil evapotranspiration.

### Bayesian likelihood function and uncertainty estimation

**Fig 3:**
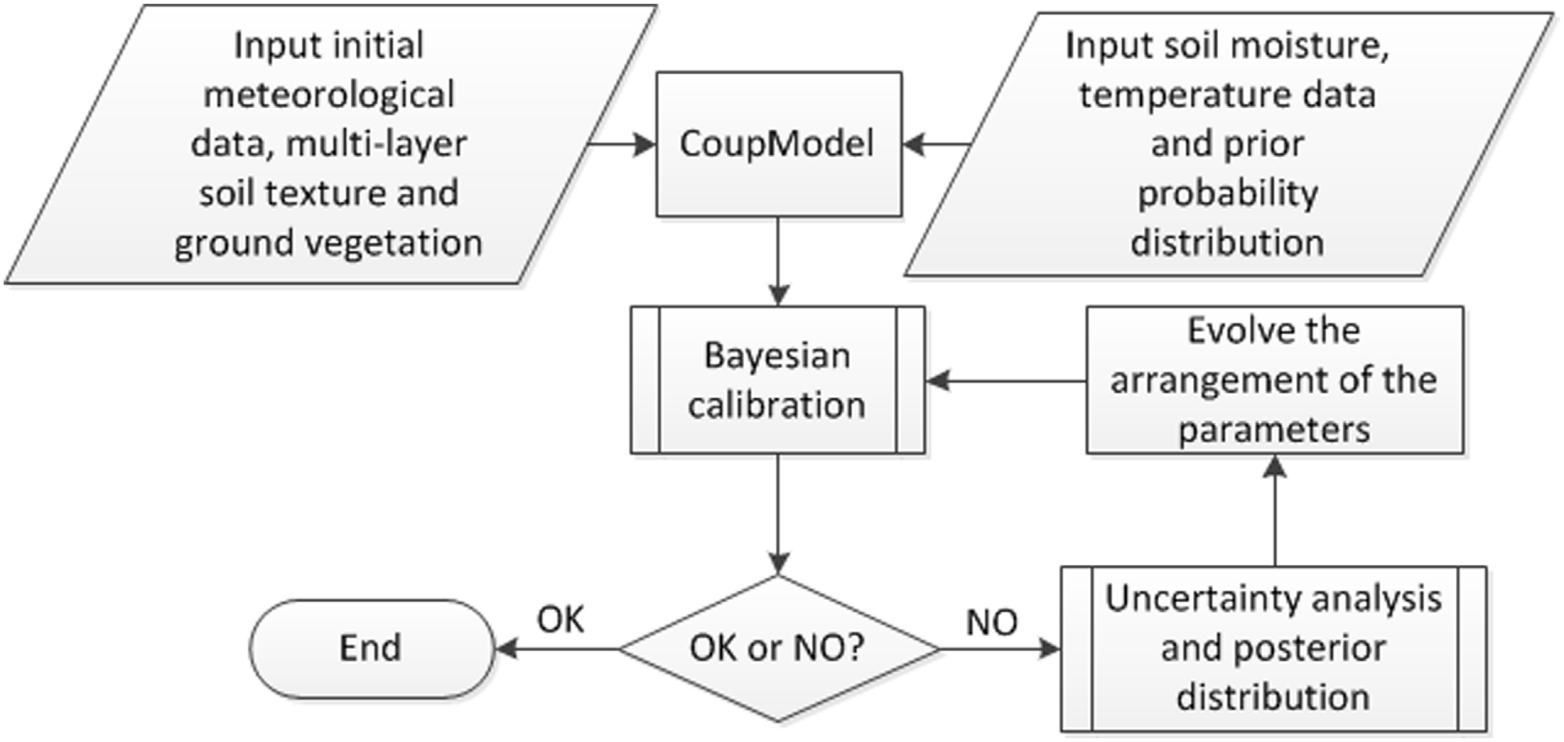
Flow chart of the evolving array of the parameters in Bayesian uncertainty estimation.

The Bayesian likelihood function is based on a more robust function than the traditional Gaussian [8], and it is one of the routine calibration methods in the CoupModel. Instead of finding the best parameter values by comparing the output to observation data, the Bayesian method determines a probability distribution in the form of a mean vector and a variance matrix for parameters. The parameter ranges will be adjusted repeatedly until the distributions for all parameters are approximately normal (Fig 3).

### Model experimental design

Parametrization and model structural uncertainty stemming from parameter and measurement uncertainty are significant challenges for the application of a physical-process-oriented model [8]. Therefore, we calibrated the model before we simulated a forecast. We used a Bayesian algorithm to determine the ability of model parameters to contain prior information in the calibration processes [26]. The prior probability distribution of the parameters was based on our observations and literature (details can be found in S2 File).

Eight layers of soil moisture and temperature observations, and 101 parameters were selected for model calibration (details in S2 File). In theory, processes and modules in the model are linked to each other and all parameters should be calibrated together [8]; however, to be more efficient, we pre-classified the parameters into two categories before model calibration: global parameters (parameter value that affects all the soil profiles), and layered parameters (parameter value that only affects the specific soil profile). In the calibration, global parameters were calibrated before the layered ones. Number of global parameter and layered parameters were 11 and 64 for the soil moisture and 10 and 16 for the soil temperature. Commonly in model construction, the relationship between climate factors and soil temperature is much simpler than that of soil water content. Consequently, soil temperature was calibrated before soil moisture calibration.

### Model evaluation

Objective functions of model evaluation can take any shape [27], and in this study, we calculated the 95% prediction uncertainty (95PPU) of Bayesian calibration distribution. Calibration and validation of the model were analyzed with the Pearson’s correlation coefficient (R) between simulated values and the observed data, and statistics were analyzed with SPSS (Version 19.0 for windows, SPSS Inc., USA). R is a robust indicator of the correlation between predicted and observed values, and it is insensitive to deviation. Because we were concerned only with the change between the pre-uncertainty and post-uncertainty analysis, we viewed R as an appropriate indicator.

## Results

### Initial model performance

The four upper soil layers exhibited satisfactory performance in the calibration of soil moisture, while the lower soil layers did not (Table 3). Soil temperature exhibited coefficients bigger than 0.75, representing a strong correlation between the simulation and observation.

**Table 3:** Statistical results of soil moisture and temperature simulated for different soil layers (before uncertainty analysis).

Layer	2013				2009			
	Water Content		Soil Temperature		Water Content		Soil Temperature	
	Pearson Coef.	Sig. (2-tailed)	Pearson Coef.	Sig. (2-tailed)	Pearson Coef.	Sig. (2-tailed)	Pearson Coef.	Sig. (2-tailed)
Layer_10	0.585**	0.000	0.961**	0.000	0.648**	0.000	0.818**	0.000
Layer_20	0.837**	0.000	0.981**	0.000	0.863**	0.000	0.920**	0.000
Layer_30	0.974**	0.000	0.986**	0.000	0.873**	0.000	0.910**	0.000
Layer_40	0.924**	0.000	N	0.000	0.659**	0.000	0.913**	0.000
Layer_50	0.664**	0.000	0.986**	0.000	-0.043	0.597	0.916**	0.000
Layer_60	0.307**	0.000	0.986**	0.000	-0.085	0.300	0.890**	0.000
Layer_80	-0.191*	0.012	0.987**	0.000	0.056	0.499	0.914**	0.000
Layer_100	-0.221**	0.003	0.986**	0.000	0.625**	0.000	0.912**	0.000

**: Correlation is significant at the 0.01 level (2-tailed).

*: Correlation is significant at the 0.05 level (2-tailed).

N: missing temperature data at 40 cm depth. Sig.: significant for two-tailed. Details and data used are described in the S3 File.

Pearson correlation coefficients of validation were approximately 0.0 for soil depths 0.5 to 0.8 m, indicating that observation and simulated data had no similarity in the trend of time series. A strong relationship was exhibited for the calibration of other soil layers, and for validation of soil temperature for all the layers.

Consequently, the model represented the upper soil layers less ideally than we had expected. The sources of uncertainty are explicitly analyzed in the following section.

### Uncertainties of input and output data

#### Input data evaluation

Uncertainties in input variables may be due to errors of sampling, measurement, or artificial in-filling of missing values. These errors can be evaluated prior to the calibration by analyzing acquisition instruments and procedures. Precipitation is the essential forcing input in the model set up; in our study, precipitation errors are represented using precipitation multipliers sampled from an uncorrelated lognormal distribution Renard, Kavetski [28] and standard deviation of each measurement is set to be 30% of the mean value treating errors in an additive manner [29].

The error model used by Huard et al. [30] portrayed hourly rainfall errors with a normal distribution *σ*^2^_*δr*_.

$$\sigma_{\delta r}=0.15r+0.2$$ (7)

Where *r* denotes precipitation (mm), the fixed component is confirmed by the resolution of rain gauges; 0.254 mm was used in our experiment after Habib et al. [31] and Huard et al. [30]. The standard variation of the daily default volume bucket gauges is estimated by $\sqrt{24}*0.254\approx 1.2$ mm; the proportional component (15%) denotes commensurability and measurements errors such as wind-induced, and rainfall and evaporation losses. Once aggregated to daily values, the proportional component is about 3% [30]. Normal distribution assigned non-zero probability value to negative rainfall, hence, we used a truncated normal distribution N:

$$P\left(\tilde{r}|r\right)=N(\tilde{r}|0.97*r,0.03*r+1.2mm,0,\infty )$$ (8)

Where $P\left(\tilde{r}|r\right)$ is a statistical distribution that is the probability of measuring an input series $\tilde{r}$ (input error model: *P*_*in*_) knowing the true input series *r*. *N* is the probability density function which can be described by:

$$N=(x|\mu ,\sigma ,a,b)=\frac{\varphi \left(\frac{x-\mu }{\sigma }\right)}{\Phi \left(\frac{b-\mu }{\sigma }\right)-\Phi \left(\frac{a-\mu }{\sigma }\right)}$$ (9)

Where *x* is a normal distribution bounded by the interval [*a*, *b*], Φ and *φ* are the cumulative probability density function and the probability density function of standard normal distribution.

**Fig 4:**
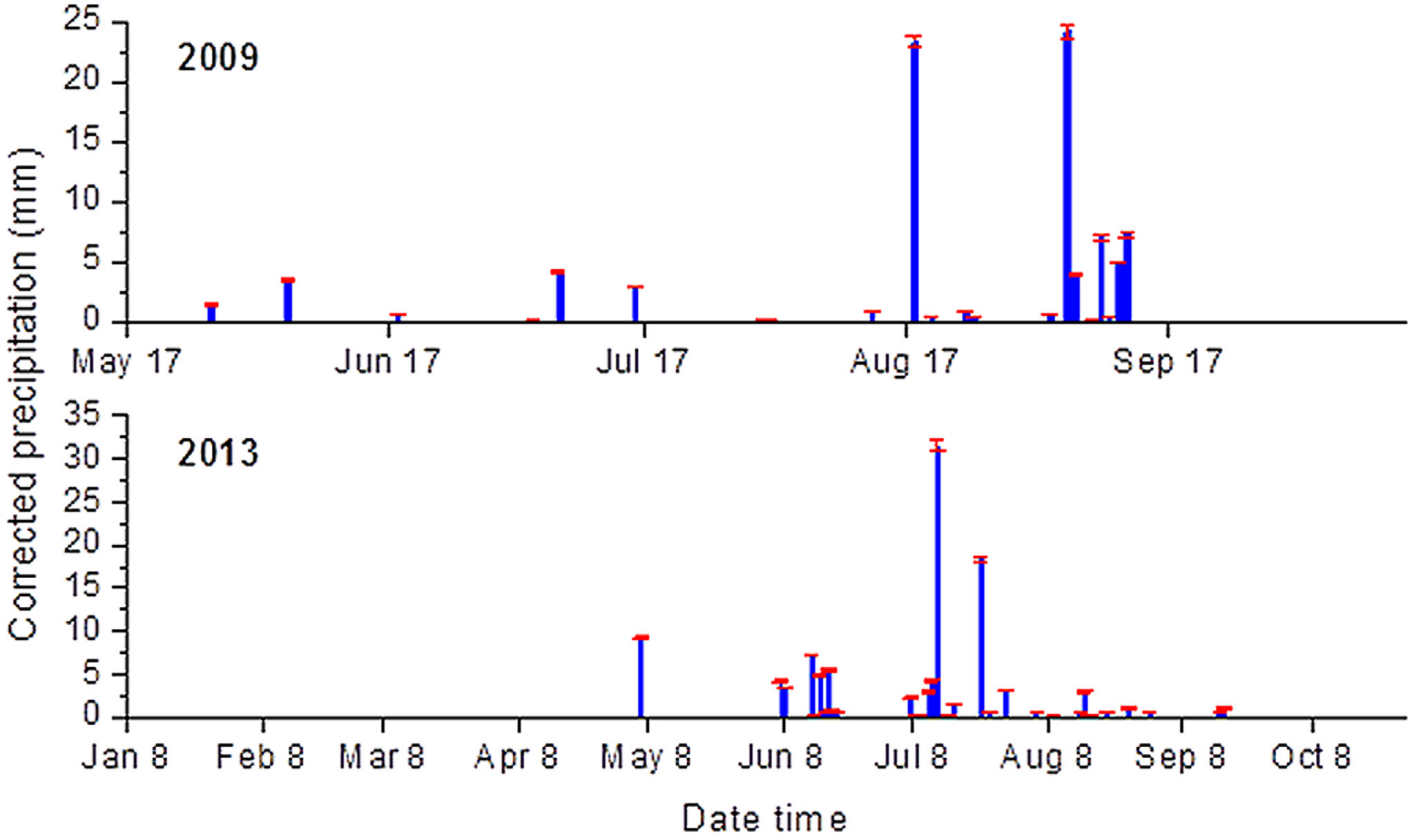
Corrected results of precipitation data. Blue bars denote inferred true precipitation (mm), and the red error bars indicate standard deviation between the records and corrected precipitation. Details and data used can be found in S1 File.

An abundance of null values prevented us from representing the error probability distribution, therefore standard deviation was used to show the deviation between the precipitation records and true values (Fig 4). The maximum standard deviation (sd.) was ± 0.67 mm and the mean sd. was ± 0.07 mm (details in S4 File).

#### Output data evaluation

**Fig 5:**
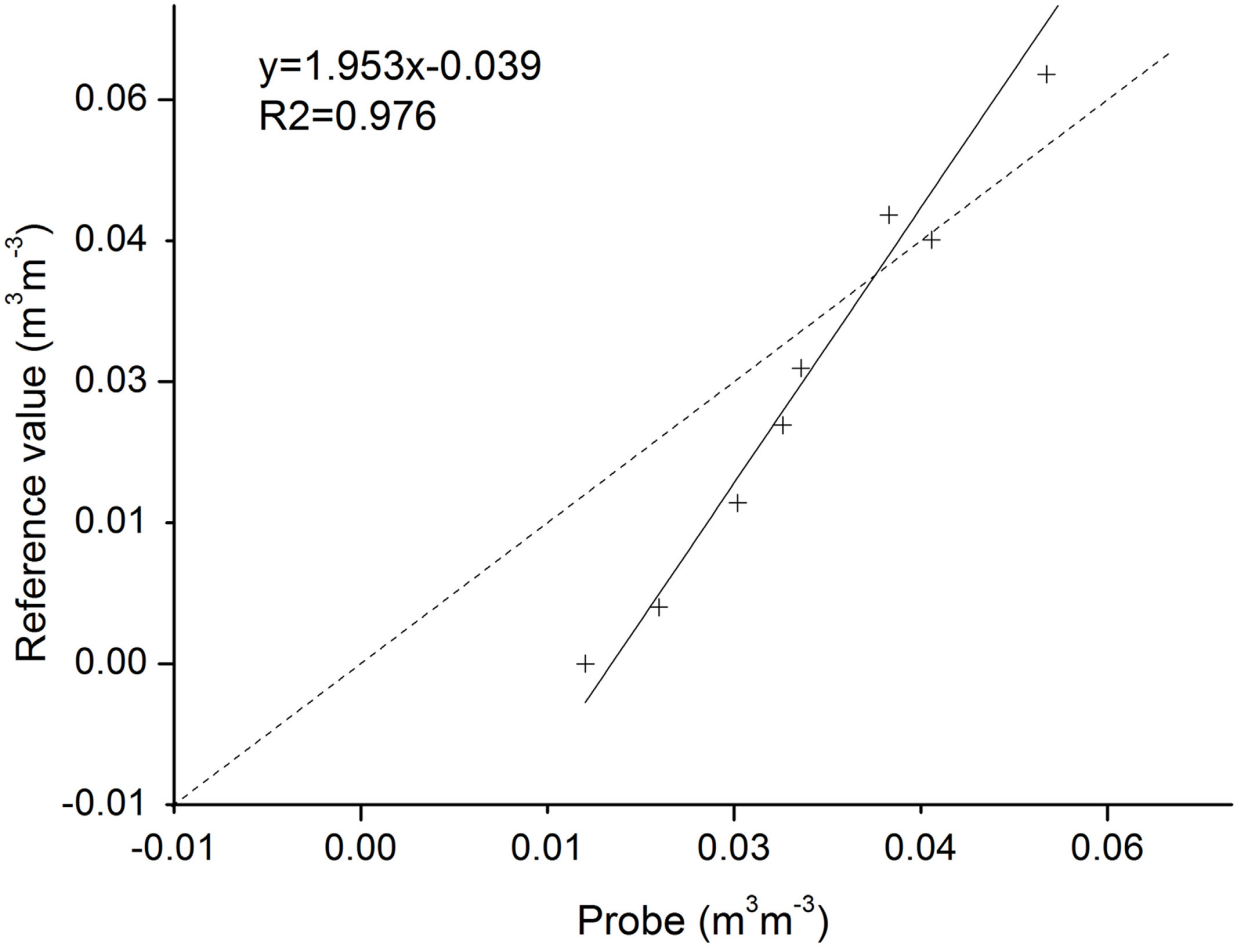
Correlation between probe and weighted values derived from measured probe and in situ weighting method. Details and data used are described in S4 File.

The major output variable that we concentrated on was soil moisture content. According to experiments of Topp et. al. [32], accuracy of TDR probes with a length of more than 0.1 m falls within sd. of ± 0.02 m^3^m^-3^ in the field, and with a length of 0.05 m, within sd. of ± 0.037 m^3^m^-3^. Therefore, we assumed that our soil moisture probe was accurate between ± 0.02 m^3^m^-3^ and ± 0.037 m^3^m^-3^. Hence, before recalibration, we evaluated error of the measured records from gravimetric sampling (Fig 5). We calculated the reference volumetric soil moisture using dry bulk density and in-situ weighted vertical soil water content. The probe records and reference data showed a high positive linear correlation (R^2^ = 0.976), with intercept of -0.039 m^3^/m^3^. Measured data and reference data had a good rectifiable correlation.

### Uncertainties of the simulation

**Fig 6:**
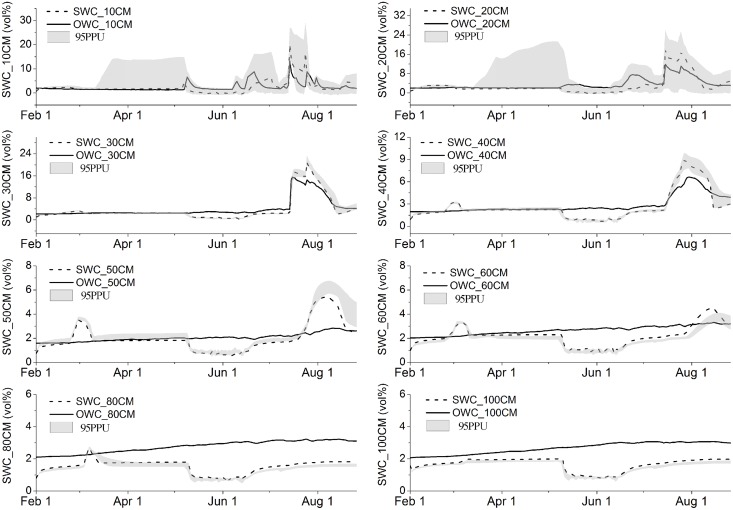
Soil moisture prediction results for Bayesian and 95% confidence interval (lighter gray) for 2013. OWC = observed soil moisture, SWC = simulated soil moisture. Details and data used are described in S4 File.

**Fig 7:**
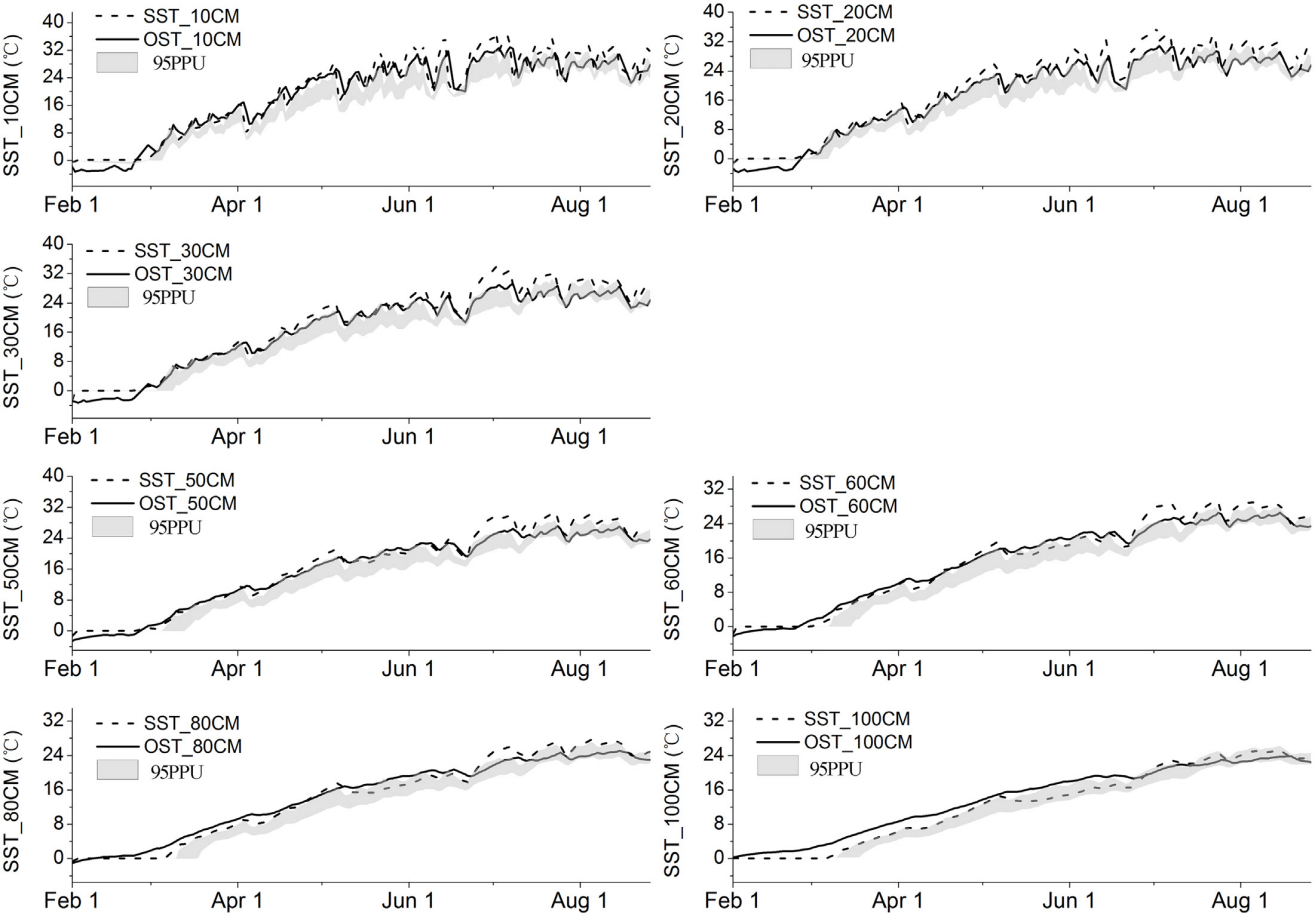
Soil temperature prediction results for Bayesian and 95% confidence interval (lighter gray) for 2013. OST = observed soil temperature, SST = simulated soil temperature. Details and data used are described in S4 File.

In this section, we demonstrate the applicability of the Bayesian approach to the identification and estimation of model performance and associated uncertainty bounds. Even with a reasonable model structure and sufficient observation data, we still could not ensure an optimal simulation. We calculated 95PPU at the 2.5% and 97.5% levels of the cumulative distribution of the output variable to look for a reasonable range of parameters; we did this through the Markov-chain Monte Carlo (MCMC) sampling, disallowing for 5% of the poor simulations (gray areas in Figs 6 and 7).

In this section, all the uncertainty in soil moisture and temperature prediction was attributed to parameter estimation. As described earlier, 75 parameters were selected for soil moisture and 26 for soil temperature calibration. The distributions were generated using 5,000 samples of 25 Bayesian random seeds; here a sample denotes a one-time random sampling of the parameter, and a seed means a one-time sampling of all parameters selected (one-seed, one-dimension). We learned that, first, 95PPU prediction uncertainty bounds for the surface and sub-surface soil moisture captured most of the observations (56% for soil moisture and 82.86% for soil temperature); this indicated that parameters have been given a sound value of prior distribution. Second, 95PPU uncertainty bounds for the lower three soil layers barely captured any observations (9.14%, 1.14%, and 0.00%, respectively). This was an indication of uncertainty in the structure of the model, in the forcing of data used in the model, or in both [33].

### Uncertainties of parameters

Bayes’ framework offers a straight-forward method for updating of likelihood distribution of parameters. When the model was calibrated for the first time, we derived the prior probability distributions of the parameters from the literature or field observation. However, after the calibration, posterior probability distributions of the parameters can be, and were used as the prior in the next calibration round. The natural Bayesian mechanism provides a sequential “training” of the model; the order of the data is irrelevant, and the posterior is the same as long as in the prior round have been processed [29].

**Fig 8:**
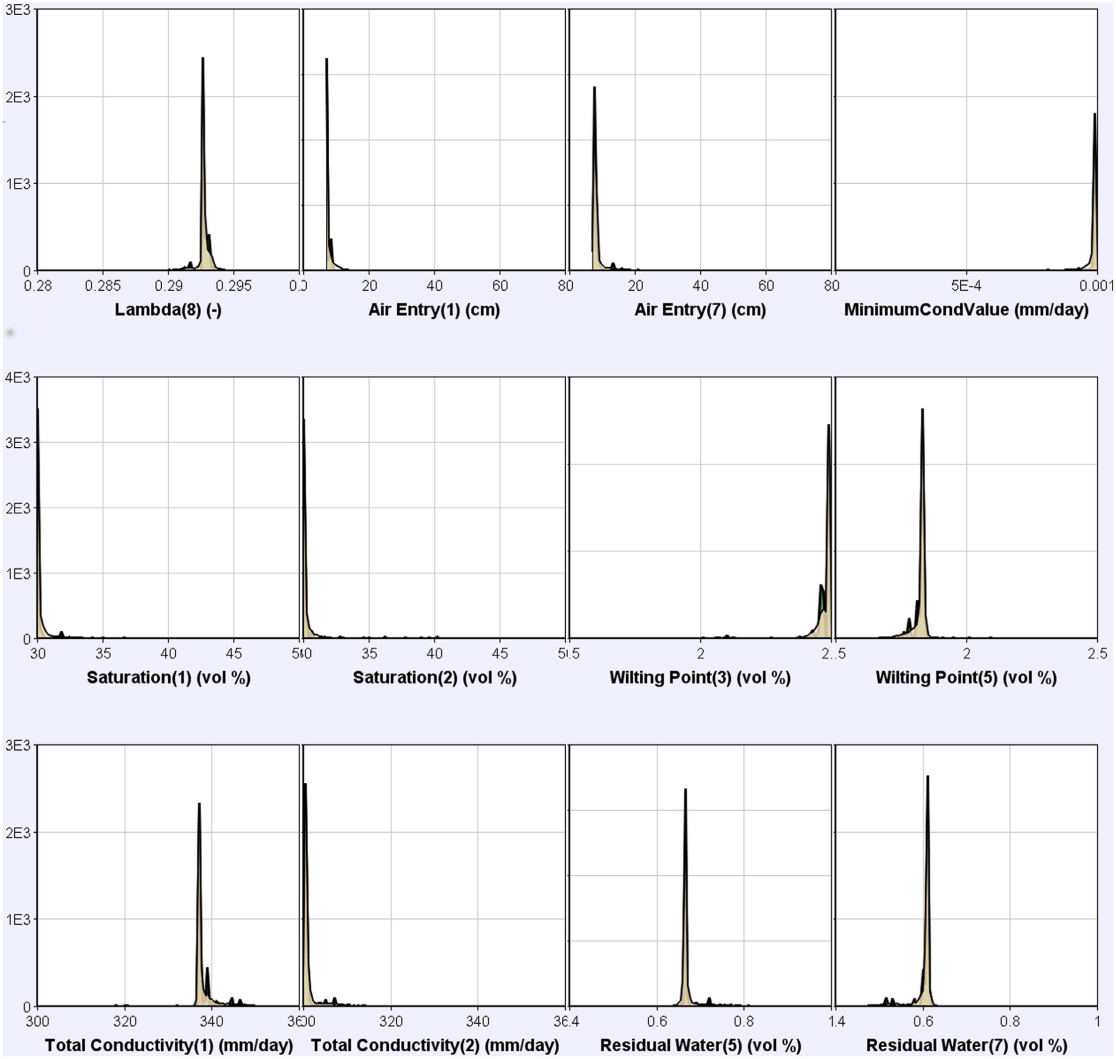
Frequency posterior probability distributions of partial parameters generated with the Bayesian approach, using 5,000 samples. Note: *Lambda(8)* was pore size distribution index at 100 cm soil depth. *Air Entry(1)* and *Air Entry(7)* were the air entry pressure at 10 and at 80 cm soil depth, respectively. *MinimumCondValue* was the minimum hydraulic conductivity in the hydraulic conductivity function; *Saturation(1)* and *Saturation(2)* denoted water content at saturation in the 10 and 20 cm soil layer, respectively. *Wilting Point(3)* and *Wilting Point(5)* meant water content at wilting point in the 30 and in the 50 cm soil layer, respectively. *Total Conductivity(1)* and *Total Conductivity(2)* implied water content at saturation in the 10 and 20 cm soil layers, respectively. *Residual Water(5)* and *Residual Water(7)* were residual soil moisture at 50, and 70 cm soil depth, respectively. Details and data used are described in S4 File.

We used the posterior probability distribution of the 12 parameters which had an irregular distribution in the model calibration to identify the uncertainty of parameter values. Sampling distributions of the parameters were highly concentrated at one point and covered a very small range of a predefined parameter range (Fig 8); this revealed that the parameter had only a few degrees of freedom. Air entry pressure at 10 and at 80 cm soil depths, wilting point at 50 cm, water content at saturation at 10 cm, and residual soil moisture at 70 cm soil depth exhibited a one-sided posterior distribution; this may indicate either a structural uncertainty of the model, or other sources of model uncertainty which were not studied here [33]. In addition, the minimum hydraulic conductivity in the hydraulic conductivity function and wilting point at 30 cm soil depth were close to the upper boundary of the observed/reported of probable parameter range [28]. Water content at saturation at 10 and 20 cm soil depths, and total conductivity at 20 cm appeared opposite of each other; this revealed that we were able to readjust the range of these parameters.

### Results after considering uncertainty of model calibration

We evaluated model performance to demonstrate uncertainties discussed above after in-filling of the missing soil temperature records, correcting the original precipitation and soil moisture, setting the first month of the simulation as the “warm-up” period, and considering parameter-bound deviation.

As expected, the results of soil moisture content simulation provided better performance either in calibration year 2013 or validation year 2009, particularly at profile depth of 60 to 100 cm (Table 4). However, validation of soil moisture at the same soil depth did not perform as well as the conventional criteria for model use had indicated. Assuming that data used in the model and value ranges of the parameters used were hypothetical, then the sole probability of uncertainty is the model structure. Soil temperature also exhibited an improvement both in the calibration and the validation year. The final results can be used in field situations.

**Table 4:** Statistical results of model calibration and validation after uncertainties analyzing.

Layer	2013				2009			
	Water Content		Soil Temperature		Water Content		Soil Temperature	
	Pearson Coef.	Sig. (2-tailed)	Pearson Coef.	Sig. (2-tailed)	Pearson Coef.	Sig. (2-tailed)	Pearson Coef.	Sig. (2-tailed)
Layer_10	0.660**	0.000	0.961**	0.000	0.650**	0.000	0.853**	0.000
Layer_20	0.837**	0.000	0.987**	0.000	0.897**	0.000	0.943**	0.000
Layer_30	0.963**	0.000	0.987**	0.000	0.976**	0.000	0.950**	0.000
Layer_40	0.907**	0.000	0.988**	0.000	0.792**	0.000	0.941**	0.000
Layer_50	0.669**	0.012	0.988**	0.000	0.098	0.290	0.928**	0.000
Layer_60	0.642**	0.000	0.988**	0.000	0.182*	0.047	0.923**	0.000
Layer_80	0.593**	0.012	0.989**	0.000	0.237**	0.010	0.888**	0.000
Layer_100	0.571**	0.003	0.990**	0.000	0.575**	0.000	0.906**	0.000

**: Correlation is significant at the 0.01 level (2-tailed);

*: Correlation is significant at the 0.05 level (2-tailed); 2013 model calibration day number: 212, 2009 model validation day number: 152

Details and data used are described in S3 File.

## Discussion

In this study, we try to investigate the components of uncertainties in ecohydrology modeling in arid regions. Implications of this finding apply to data quality and modeling as discussed below.

Sun and wind increase evapotranspiration, therefore soil moisture at 10 cm soil depth in our study was less than that at deeper layers; it also responded to rainfall fastest. However, soil water at 20 to 40 cm soil depth is critical to the physiological processes and the survival of the shallower plants in arid and semiarid areas [34, 35]. Soil moisture between 50 and 100 cm depth remained about constant in the course of the study because it is barely affected by plant roots and climatic conditions this far from the ground surface. Our results also indicated that the most difficult soil portion in which to simulate was the deepest studied (from 50 to 100 cm). As we supposed, soil water content in these layers can hardly be influenced by precipitation (because maximum precipitation event could not reach this depth of the soil profile), and soil moisture was too low (about 2% cm^3^cm^-3^) to be captured by previous ecosystem models.

Validation results performed somewhat poorly in soil moisture modeling; before model validation, the parameter set was consistent with the initial environmental condition of the calibration period [36, 37], and initial environmental parameters should be recalibrated when they are used for model validation [38]. In practical application, however, no more adjustments were done before model validation, therefore, the results of validation were less than desired.

### Implications for data quality

To deal with missing observations (precipitation with non-wind interference) in input data, methods can be adopted such as probability algorithm, random multiplier, and an estimation function [28, 30, 33]. An estimation function of precipitation was used in this study, with an apparent effect especially in heavily rainfall events (increased by 0.95 mm for 2013 and 0.72 mm for 2009). We concluded that modification of measuring data is an important factor in uncertainty of the simulation.

The accuracy of TDR was an issue in model simulation. Although the precision of the equipment is satisfactory (with sd. of about 2–3% cm^3^cm^-3^) to most soil moisture measurements (value is about 10–30% cm^3^cm^-3^), measurements in environments where soil moisture is about 2–3% acquire a deviation (about 2% volumetric soil moisture) that is an important component of error.

### Implications for modeling

First month of calibration for soil temperature was an indicator for model “warm-up” stage (Fig 7). This procedure allows finding a reasonable initial value range. Our results indicated that a “warm-up” period of the model was imperative for soil water and soil temperature calibration. Hence, a “warm-up” period should be established and confirmed by the modeler in model calibration. Sufficiently-long sequence of the observation data is critical for the use of the model, and the length should be synchronous with the time-step of the model.

Ajami, et al. [33] suggested that ignoring either input driving error or model structural uncertainty will lead to unreasonable uncertainty intervals and unrealistic model simulations. Each ecohydrological model has its own precision and theoretical minimum of soil moisture. In this application, we could not verify whether the CoupModel has the ability to predict soil moisture for extremely low soil moisture conditions of arid or semiarid regions. Our results demonstrated that the CoupModel can provide a satisfactory simulation of soil moisture above and below 40 cm depth even if it cannot capture the true situation.

Ephemeral vegetation is another vital factor in the balance of soil moisture in arid regions. For example, shortly after an extreme rainfall event many ephemeral plants appear on the ground surface. Therefore, equilibrium in soil moisture changes easily in arid regions; with a new equilibrium state, simulation of surface soil moisture in arid regions is more difficult than in other regions. Furthermore, surface sand is easily removed by wind in this sparsely vegetated area, changing soil texture readily. Therefore, vegetation and soil texture database from arid regions may be variable between different years, resulting in a new uncommon source of model uncertainty in arid regions.

We evaluated the simulation after considering all probability factors together rather than one-at-a-time as was done in previous work [30], this can exclude interplay which could be generated among the different error sources of the ecohydrology model. However, this approach did not allow us to attribute the predictive uncertainty to individual sources.

An understanding of ecohydrological processes is one of the critical factors in model building, and different ecosystems need different module combinations. As discussed above, the modeler needs to consider ephemeral vegetation and drifting sand in surface-soil moisture modeling in an arid region after an extreme rainfall. A profound understanding of the relationship between the ecosystem and the model can reduce the uncertainties of model structure.

## Conclusions

Modeling of soil hydrological processes is critical to ecohydrological simulation in arid regions. We demonstrated uncertainties from input and output data, parameter value array, and model structure. We concluded that the performance of the CoupModel was satisfactory for moisture simulation at soil depths between 0 and 40 cm, and excellent for soil temperature at < 1 m depth; however, simulations for soil mean deeper than 40 cm were not adequate. Therefore, the CoupModel can be effectively utilized for ecohydrological modeling for surface soils in arid regions.

Parameter uncertainty dominated the principal uncertainty for modeling of the top 40 cm soil moisture, and structure uncertainty was the governing factor at soil depth above 40 cm. Moreover, ephemeral vegetation and drifting sand were potential impact factors on model stability. Analysis of model uncertainty helps us detect problems in the simulation, and promotes model applicability to new areas. Consequently, UA is a necessary step in the utilization of models.

In arid regions of China, numerous DOEs have been vegetated since the year 2000 with sand-stabilizing plants to stop desertification. These efforts can be made more successful and efficient by understanding soil moisture relationships. Simulation of soil moisture in arid regions is a foundation for decision-making about the type of vegetation that can be used for sand stabilization, and prediction of the area of cultivation in the DOE. Simulation of soil moisture can serve as preparation for the estimation of soil water carrying capacity in arid and semiarid regions.

## Supporting Information

S1 File Dataset of the forcing data and the validation data in the model set up. (XLSX)

S2 File Dataset of the parameters used in the model calibration and uncertainty analysis. (DOCX)

S3 File Dataset of the Tables 3 and 4. **: Correlation is significant at the 0.01 level (2-tailed). *: Correlation is significant at the 0.05 level (2-tailed). N: missing temperature data at 40 cm depth. Sig.: significant for two-tailed.
(XLSX)

S4 File Dataset of the Figs 2 and 4 to 8. OWC = observed soil moisture, SWC = simulated soil moisture; OST = observed soil temperature, SST = simulated soil temperature; Lambda(8) was pore size distribution index at 100 cm soil depth. Air Entry(1) and Air Entry(7) were the air entry pressure at 10 and at 80 cm soil depth, respectively. MinimumCondValue was the minimum hydraulic conductivity in the hydraulic conductivity function; Saturation(1) and Saturation(2) denoted water content at saturation in the 10 and 20 cm soil layer, respectively. Wilting Point(3) and Wilting Point(5) meant water content at wilting point in the 30 and in the 50 cm soil layer, respectively. Total Conductivity(1) and Total Conductivity(2) implied water content at saturation in the 10 and 20 cm soil layers, respectively. Residual Water(5) and Residual Water(7) were residual soil moisture at 50, and 70 cm soil depth, respectively.
(XLSX)
