# Supplementary material for: Uncertainty in Ecohydrological Modeling in an Arid Region Determined with Bayesian Methods
Source: PLoS One. 2016 Mar 10;11(3):e0151283. doi: 10.1371/journal.pone.0151283 (PMC4786118; doi:10.1371/journal.pone.0151283)
Supplement: S2 File — (DOCX) [file pone.0151283.s002.docx]

**Parameters used in the model:**

lambda: 0.280~0.296

air entry: 55~68

saturation: 45%

wilting point: 1.5~2.5

residual water: 0.5

macro pore: 3~10

Matrix Conductivity: 200

Total Conductivity: 331~1000mm/d

DirectThroughfall: 0.8

EPMaxRate: 15mm/d

HumRelMean：43.13%

minimumcondvalue：1.0e+14~1.0e+3

TempFacAtZero：0.45~0.55

TempFacLinlncrease：0.023

RoughLBareSoilMom: 0.0005902(Per)

DVapTortuosity：0.66

InitialGroundWater：-5

CritThresholdDry：118.6

DemandRelCoef：0.1~1.9/day

n Tortuosity: 0.5~2.5

Albedo: 25~30%

EquilAdjustPsi: 3.3894(Per)

Brooks Corey Function.

air entry: 13.3±5 cm

saturation: 41±9 (Vol%)

wilting point: 10±3 (Vol%)

residual water: 6.5±0.6 (Vol%)

Total Conductivity Sandy loam: 1060.8±200 mm/d

Other parameters which don’t involved here use the default value.
